# Donkey Skin Trade and Its Non-compliance With Legislative Framework

**DOI:** 10.3389/fvets.2022.849193

**Published:** 2022-03-17

**Authors:** Yuri Fernandes Lima, Patricia Tatemoto, Emily Reeves, Faith Adelaide Burden, Eduardo Santurtun

**Affiliations:** ^1^The Donkey Sanctuary, Mexico City, Mexico; ^2^The Donkey Sanctuary, Sidmouth, United Kingdom

**Keywords:** animal welfare, animal health, biosecurity, donkey, *ejiao*, ethics, sustainability

## Abstract

Donkeys (*Equus asinus*) are facing a global crisis. Their welfare, and even survival, is being compromised as the demand for their skins increases. This demand is driven by the need to supply raw materials to produce *ejiao*, a Traditional Chinese Medicine made from collagen extracted from donkey skins. Since there is no productive chain for donkey skin production outside of China, the global trade is an entirely extractive industry that has resulted in the decimation of some local donkey populations. The donkey skin trade is demonstrably unsustainable, from the ethical issues associated with poor welfare, to the biosecurity and human health risks the trade poses; and it violates both legal frameworks and moral expectations at both a national and global level.

## Introduction

The increasing demand for donkey skins results in large-scale handling, transport, and slaughter of donkeys, and is severely compromising their welfare, and even survival in some local areas. It is driven by the production of *ejiao*, a traditional Chinese remedy that some people believe has medicinal properties. Most countries do not farm donkeys, and the trade therefore relies on animals that are traded both legally and illegally. In some instances, donkeys are gathered on “fake farms,” increasing the risk of disease transmission, until the numbers are sufficient to warrant transport to slaughterhouses (1).

To be considered sustainable, a production system should be acceptable not only in the present, but into the future (2, 3) and should consider the availability of resources, the impacts caused by the system, and the ethical implications of action (4). An extractive trade in an animal whose population is rapidly depleting, and which gives rise to both biosecurity and ethical concerns cannot be described as sustainable (2–5).

Donkeys are often mistreated during all stages of the trade, from collection to slaughter (1, 6). They may spend many hours confined during long journeys, and often do not have access to adequate water or food, or to any veterinary care during those journeys (7). They are also routinely held without food or water prior to slaughter. Inside the slaughterhouse, in the absence of much scientific knowledge and specific slaughter guidelines, it is likely slaughterhouse workers are failing to correctly apply the techniques and tools required to ensure humane slaughter. When stunning is used as a technique for humane slaughter, it must result in immediate loss of consciousness (8) and, as there is currently little scientific knowledge specifically for stunning donkeys, it is likely that some of the practices employed in slaughterhouses are outside of national laws that govern the humane treatment and welfare of animals.

Animals are sentient beings, with widely recognized cognitive abilities (9) and animal mistreatment and/or neglect is a crime in many countries. Studies have shown that animals are able to experience pain and suffering in a similar way to human beings (10–12), and there is growing and irrefutable confirmation of the cognitive and neurophysiological complexity of non-human animals. To develop high quality guidelines that guarantee good animal welfare, it is necessary to recognize the complexity of other animals' neurological systems.

Additionally, the lack of traceability within the global skin trade creates a high biosecurity risk (1). The OIE (13) defines biosecurity as: “set of management and physical measures designed to reduce the risk of introduction, establishment and spread of animal diseases, infections or infestations to, from and within an animal population.” It is widely known that chronic stress may lead to immunosuppression, and the effects of stress compounds factors such as inadequate access to food and water, making donkeys highly susceptible to becoming vectors of disease (7). The diseases which may be carried by donkeys include zoonotic diseases such as glanders (14), a highly contagious infectious disease that is often fatal in both animals and humans. The risk is exacerbated by the movement of animals without sufficient identification and traceability. The lack of traceability in the donkey skin trade not only represents a serious threat to public health, but it also results in the violation of laws and regulations designed to ensure the safety of both the human and animal population.

In addition to national laws, the practices involved in the donkey skin trade also violate international agreements and ethical standards. At a national level, the trade often defies laws governing the treatment of animals, biosecurity, and environmental protection (7). At the international level, the global donkey skin trade breaches numerous rules of World Organization for Animal Health (OIE) including the requirements for disease control and the duty to guarantee animal health and welfare; provide veterinary assistance; and ensure that staff are trained to work with the relevant species.

In the OIE Terrestrial Animal Health Code (Chapter 7.3), on the transport of animals by land for example it does not specify requirements for donkeys but makes clear recommendations regarding the transport of other equines, such as horses (15). On the other hand, the OIE Terrestrial Animal Health Code has established the responsibilities toward working equids (Chapter 7.12 - welfare of working equids).

In the same sense, the Council of the European Union (article 1.4.c. of chapter V of Regulation n° 1/2005) establishes that “domestic Equidae can be transported for a maximum of 24 h. During the journey, the animals must be watered and, if necessary, fed every 8 h.” It also stipulates that “the lack of an adequate level of animal welfare is often due to a lack of training. Therefore, any person handling animals during transport must have undergone training, provided only by bodies accredited by the competent authorities” (16).

The inherent welfare implications and challenges associated with the global skin trade necessitate a discussion of the ethical and legal frameworks being infringed. Ethical concerns and scientific knowledge contribute to the development of legislation that has the power to ensure that practices related to animal health and welfare meet social expectations. Regardless of the existence of legislation, the public play an important role in ensuring their expectations pertaining to animal welfare are met and they have increasing opportunities to influence the way animals are treated. However, access to information is essential for people to make decisions, and call for regulations, that align with their ethics. For this reason, the ethical issues related to the skin trade have both legal and public expectation dimensions, and both are required to address issues of cruelty and unsustainability within the trade. This study aims to shed light on legislative frameworks of the global trade in donkey skins.

## Animal Welfare Legislation

Many countries have legal frameworks governing animal welfare and, while the existence of legislation does not necessarily guarantee that mistreatment will not occur, it establishes a first step that guides regulation, and often reflects the ethical expectations of a society.

The methods of rearing farm animals described in the book Animal Machines (17) led the British government, under the guidance of zoologist and professor Roger Brambell, to create a scientific committee known as the Brambell Committee, which issued the *Brambell Report* in 1965 making some recommendations and providing that animals must have five basic freedoms being the freedom from hunger and thirst; the freedom from discomfort; freedom from pain, injury or disease; freedom from fear and distress; and the freedom to express natural behavior. These freedoms have been considered one of the international benchmarks for animal welfare and have been adopted by many countries. Other countries have moved beyond the Five Freedoms and work with a Five Domains Model that was updated in 1994 considering the latest animal welfare science evidence of: (1) Nutrition, (2) Physical environment, (3) Health, (4) Behavioral interactions, and (5) Mental state (positive and negative). The Model emphasizes what matters to animals in welfare terms of their subjective experiences (affects) and the interactions of affects with physiological mechanisms (18).

One of the first pieces of animal welfare legislation is known as Martin's Act. In 1822, Richard Martin, an Irish Member of Parliament, “The Cruel Treatment of Cattle Act” (19). This Act was repealed by the Cruelty to Animals Act 1849, which reiterated the offenses of beating, ill-treating, over-driving, abusing and torturing animals (20).

Animal abuse is not tolerated in many societies and this trend can be considered aligned with the increasing evidence of the sentience, and even consciousness, of non-human animals, as discussed in “The Cambridge Declaration on Consciousness” (9). A growing number of European countries, including Austria (1988), Germany (1990), Switzerland (2003), France (2015), and Portugal (2017), (21), no longer consider non-human animals as objects.

The current EU animal welfare policy has been elaborated on, and supported, by the “Protocol on Protection and Welfare of Animals,” via the Treaty of Amsterdam in 1999 (22). This document recognizes animals as sentient beings and obligates European institutions to address animal welfare requirements when formulating legislation in diverse areas such as agricultural, transport, market, and research (22).

This trend is echoed outside of Europe, for example, the New Zealand Animal Welfare Amendment Act (No. 2 of 2015) recognizes animal sentience (23); the Indian Ministry of Environment and Forests declared in 2015 that all cetaceans are non-human persons; Colombia's 2016 reform of the National Animal Protection Statute recognizes animals as sentient beings and introduces new penalties for animal's abuse; and Argentina, where the chimpanzee Cecilia was declared a subject of law/rights (*sujeto de derecho*) (24).

While Brazil does not have a Federal Law recognizing animal sentience, some states including Paraíba, Rio Grande do Sul and Santa Catarina have introduced laws that change the legal status of animals.

In this context, it is essential to have scientists building high quality evidence focused on what is needed to provide good or high levels of welfare to the animals in our care. There are current, valid methods to determine what is necessary for good welfare. However, in cases where there is insufficient scientific knowledge, or inadequate measurement tools, it is necessary to make decisions-based on the precautionary principle. In relation to animal welfare, the precautionary principle provides the animals with the benefit of doubt related to potential suffering (21, 25).

## The Donkey Skin Trade—Global Context and the Case of Brazil

The global donkey skin trade is largely unregulated, often illegal, and has significant negative impacts including harm to the livelihoods of vulnerable communities, the risk of disease spread, environmental pollution, and threats to other animals (7). In several countries where the skin trade is illegal, donkey trafficking, and slaughter remain widespread. Countries such as Mali, Burkina Faso and Ghana have all prohibited donkey slaughter and the export of skins, but tens of thousands of donkeys are still being trafficked annually across the open border between these three countries for slaughter (7). In other countries the trade itself is legal but there are multiple illegal practices associated with the transport and slaughter of donkeys. The trade undermines actions taken by national governments to protect their national donkey herds (7).

In Brazil, the Federal Constitution from 1988 (article 225) raised the environment to the status of a fundamental right, entirely intended to protect the environment for present and future generations. This clause cannot be changed except by a new Constitution (26). It affords clear protections to the environment, including the non-human animals living within it. Also, the Federal Constitution of 1988 (Item VII of paragraph 1 of article 225) foresees that it is the responsibility of the Public Power to protect fauna and flora, and to prohibit practices that put their ecological function at risk, cause the extinction of species, or subject animals to cruelty (26). This is the constitutional provision of the protection of the non-human animal in the Brazilian legal system and the mechanism by which practices that subject animals to cruelty are expressly prohibited. The rule of the prohibition of cruelty to animals implicitly recognizes animal sentience and gives rise to the principle of animal dignity and the autonomy of Animal Law in Brazilian Law (21).

The extensive list of practices that are considered mistreatment includes abandoning a sick, injured, exhausted, or mutilated animal; failing to provide an animal with what is required to meet basic needs, including veterinary assistance; transporting animals on vehicles for more than 12 h, without water and food; corralling animals in such a number that it is not possible for them to move freely; and leaving animals without water and food for more than 12 h (Decree no. 24.645/1934 foresees, in article 3, Brasil, 1934). These illegal practices are frequently seen in the donkey skin trade in Brazil and occur in violation of those legislative instruments.

Regarding animal mistreatment, a Federal Law criminalizes ill-treatment of animals, providing for a prison sentence of 3 months to 1 year and a fine [Brazil Federal Law No. 9,605/1998, Environmental Crimes Law, article 32, (26)]. In response to the lenience of the penalty, and resulting impunity, there are several bills in progress in the National Congress to increase the penalty.

The legislative triad (Federal Constitution of 1988, Decree no. 24.645/1934, and Federal Law n° 9.605/1998) is what protects non-human animals, including donkeys, against cruelty and mistreatment in Brazil.

Since there is evidence of donkey mistreatment within the skin trade, the trade also violates the Decree no. 9.013/2017 (article 88), which provides that “the establishment is obliged to adopt measures to prevent the mistreatment of animals and apply actions aimed at animal protection and welfare, from departure at source to slaughter” (27).

The implications of the donkey skin trade in Brazil extend beyond breaches of animal welfare legislation. The Federal Constitution of 1988 (article 215) protects the Brazilian cultural heritage in all its expressions, ways of creating, doing, and living. Donkeys have well-documented historical and cultural importance as an integral part of Brazil's cultural heritage, according to the constitutional provision (28).

Beyond the legal sphere, there are resolutions, ordinances, and normative instructions, from bodies such as the Ministry of Agriculture, Livestock and Supply (MAPA), the Federal Council of Veterinary Medicine (CFMV), and the National Traffic Council (CONTRAN), regarding animal welfare which are also breached by the donkey skin trade.

## Neurophysiological and Cognitive Basis for Preventing Animal Suffering

The scientific community recognizes all vertebrates as sentient, that is, capable of experiencing positive and negative emotions in response to stimuli that come through their sensory system (9). Brain structures associated with emotions in humans, such as the structures that make up the limbic system, have been widely demonstrated in all vertebrates (29). The experience of pain has been scientifically demonstrated in all vertebrates (30, 31). Nociceptors, the sensory receptors that respond to painful sensations (32), have been detected in all vertebrates. Pain can be induced by tissue damage, as well as chemical, mechanical, and thermal stimulation. Such injuries result in a nociceptive response, which is transferred to the integrating system, the brain, and interpreted as pain. While pain represents a source of physical stress, fear in turn is a psychological response that represents a key mechanism that has been selected to optimize survival. Even countless species of invertebrates have neuro-anatomic-physiological structures that, at the very least, suggest their sentience (9).

The “Cambridge Declaration on Consciousness” affirmed that non-human animals are not only sentient (can experience emotions, feelings, and physical sensations like pain), but they are conscious beings. The declaration was signed by a prominent international group of twenty-six cognitive neuroscientists, neuropharmacologists, neurophysiologists, neuroanatomists, and computational neuroscientists who gathered at The University of Cambridge to reassess the neurobiological substrates of conscious experience and related behaviors in human and non-human animals (9).

Animals' capacity to experience and express emotions has also been demonstrated (10, 12, 33, 34) particularly their capacity to feel anxiety, fear (33, 35), anhedonia (36), and motivation (37). Non-human animals can also change their behavior, based on previous experience and, like humans, can be more optimistic or pessimistic due their experiences, a response called cognitive bias (38). Additionally, animals' ability to demonstrate complex cognitive skills, involving memory, learning, and assessment has been proven and, recently, more complex attributes such as fairness and morality (11, 39, 40) have been evidenced.

Studies have shown the complexity of donkeys' cognitive abilities and emotions. Donkeys have high cognitive abilities and are considered intelligent animals when comparatively scored on an analogous human scale (41). Regardless of studies in donkeys, the evolutionary mechanisms provide unequivocal evidence of the continuum of brain complexity of animals, then the differences are more related to degree rather presence of such capacities.

Despite evidence demonstrating animals' ability to think and feel, and their intrinsic value, there are countless examples of animal welfare abuses across animal industries globally. The practices that many species are subjected to, and the neglect of their basic needs, give rise to an urgent need for discussion and action. The suffering that donkeys experience in the global trade in their skins is a pertinent example of an issue on which action is urgently needed.

It is unquestionable that donkeys have interests that must be considered, and that they are sentient beings that suffer and feel pain, fear, pleasure, and herein are the ethical grounds needed to protect donkeys from mistreatment.

## Conclusion

The extractive and unregulated nature of the global donkey skin trade results in significant risks including poor animal welfare, environmental degradation, biosecurity and public human health risks, and non-compliance with national legislation in the countries in which the trade operates. Donkeys are sentient and often hardworking beings, and they experience indescribable suffering in this trade. The donkey skin trade is demonstrably unsustainable, and it violates both legal frameworks and moral expectations at both a national and global level. In response to the growing body of evidence demonstrating the donkey skin trade to be inhumane, unsustainable, and potentially unsafe, the slaughter of donkeys and export of their skins should be stopped.

## Author Contributions

YL and PT had the same contribution in writing the first draft and review. ER edited and review. FB and ES review. All authors contributed to the article and approved the submitted version.

## Conflict of Interest

The authors declare that the research was conducted in the absence of any commercial or financial relationships that could be construed as a potential conflict of interest.

## Publisher's Note

All claims expressed in this article are solely those of the authors and do not necessarily represent those of their affiliated organizations, or those of the publisher, the editors and the reviewers. Any product that may be evaluated in this article, or claim that may be made by its manufacturer, is not guaranteed or endorsed by the publisher.
